# A concordant expression pattern of fatty acid synthase and membranous human epidermal growth factor receptor 2 exists in gastric cancer and is associated with a poor prognosis in gastric adenocarcinoma patients

**DOI:** 10.3892/ol.2015.3609

**Published:** 2015-08-14

**Authors:** HE LI, XUEFEI WANG, ZHAOQING TANG, FENGLIN LIU, WEIDONG CHEN, YONG FANG, CONG WANG, KUNTANG SHEN, JING QIN, ZHENBIN SHEN, YIHONG SUN, XINYU QIN

**Affiliations:** Department of General Surgery, Zhongshan Hospital, Fudan University, Shanghai, P.R. China

**Keywords:** fatty acid synthase, human epidermal growth factor receptor 2, gastric cancer

## Abstract

Fatty acid synthase (FAS) and human epidermal growth factor receptor 2 (HER2) are overexpressed in gastric cancer (GC), and certain interactions have been found between FAS and HER2. A total of 94 patients were enrolled in the present study, each of whom underwent a D2 radical surgery in Zhongshan Hospital affiliated with Fudan University (Shanghai, China) between 2000 and 2005. The expression of FAS and HER2 was assessed by immunohistochemistry analysis of tissue microarrays generated from GC and non-tumor tissues. All data were analyzed by GraphPad Prism 5.0 to investigate the association between FAS and HER2 and to detect the potential association with prognosis. FAS (P<0.0001) and membranous HER2 (mHER2; P=0.0021) were overexpressed in the GC tissues, and a bidirectional and strong correlation was demonstrated between FAS and mHER2 in the tumor tissues. The expression of cytoplasmic HER2 (cHER2) was significantly lower in the GC tissues compared with the non-tumor tissues (P=0.0005), and cHER2 was expressed at a higher level in tumors that had better differentiation compared with poorly-differentiated tissues (P=0.0503). Patients with a concordant expression pattern of FAS and mHER2 showed a significantly poorer prognosis than the non-concordant group (P=0.0096; hazards ratio, 3.2801; 95% confidence interval, 1.5781–6.8176). GC tissues significantly overexpress FAS and mHER2 and the expression of these two markers is associated. Patients with a concordant expression of FAS and mHER2 are more likely to suffer a poor prognosis.

## Introduction

Gastric cancer (GC) is a common disease and is the second leading cause of cancer-related mortality worldwide (1). Recently, significant developments have been made in the field of cancer-specific targeted therapy, and fatty acid synthase (FAS) and human epidermal growth factor receptor 2 (HER2) have emerged as possible markers of GC (2–4).

Fatty acids (FAs), which are components of the membrane and are essential in energy production, are absorbed from foods (exogenous pathway) or synthesized from intracellular substrates and enzymes (mainly through an endogenous pathway or *de novo* synthesis). FAS is a key biosynthetic enzyme involved in *de novo* synthesis, through which long chain FAs (LCFAs) can be produced with acetyl-CoA, malonyl-CoA and NADPH as substrates (5).

FAS is expressed in the liver, lipogenic tissues, proliferating fetal cells and hormone-sensitive cells, but is generally poorly expressed by non-tumor tissues (6–9). However, it has been reported that FAS is highly expressed in several cancers, including prostate, ovarian, breast, endometrial, thyroid, colorectal, bladder, gastric and lung cancers (5,10). Moreover, this pattern of expression in transformed tissues cannot be modulated by physiological signals, as is occasionally the case in non-tumor tissues. Therefore, tumors can perpetually synthesize LCFAs to facilitate their proliferation and infiltration. At the same time, FAS is becoming increasingly significant in tumor diagnosis, prognosis and even treatment, particularly since a close association has been proposed between energy metabolism and tumor genesis (5,11). Several *in vitro* studies have demonstrated the diagnostic value of FAS in cancer (12–14) or precancerous lesions (15). However, the role of FAS in gastric carcinogenesis has not been clearly identified.

FAS expression is modulated in multiple ways in cancer cells, one of which is through transcriptional regulation. Extracellular stimulants can ultimately activate FAS gene expression through the Ras/Raf/MAPK and PI3K/Akt pathways, but numerous other factors are also important, such as HIF-1α, mTOR and SPOT14 (16–18). In general, a complicated network of molecules is involved in FAS-related carcinogenesis, including HER2.

HER2 is a type of tyrosine kinase receptor that belongs to the erbB family. Similar to FAS, HER2 has been proven to be underexpressed in normal tissues, but in a number of tumors it is abnormally overexpressed and activated, including GC where patients with overexpression of HER2 have a morbidity rate of 10–30% (19). HER2 can activate multiple downstream pathways, including the PI3K/Akt and Ras/Raf/MAPK pathways, which are the upstream signals of FAS. On the other hand, sufficient production of phospholipids for membrane microdomains will result in accommodation of receptor tyrosine kinases expressed on the membrane, including HER2 (20). Therefore, there appear to be certain significant correlations between FAS and HER2, which may synergistically modulate gastric carcinogenesis.

The roles that FAS has played in gastric carcinogenesis are under investigation. Overexpression of membranous HER2 (mHER2) in cancer tissues indicates a poor prognosis. Anti-HER2 therapy has been recommended in the treatment of HER2-positive GC patients (2), but the exact effect of this approach is yet to be determined. The experience gained from HER2-targeted therapy in breast cancer has shown that drug resistance inevitably interrupts the process of cancer treatment. Examinations of FAS and HER2 expression in breast (21,22), ovarian (20) and oral (23) cancers has been performed *in vivo*. However, few studies have investigated FAS expression or its association with HER2 in GC. In the present study, FAS and HER2 expression patterns were examined in 94 GC tissues and compared with adjacent non-tumor tissues. Finally, the expression of FAS and HER2, and their association with clinicopathological features and prognosis was examined in the GC patients.

## Materials and methods

### 

#### Ethics statement

The present study was approved by the Zhongshan Hospital Review Board (Shanghai, China), and all enrolled patients provided written informed consent to participate in the study.

#### Patients enrolled

A total of 94 patients with GC who underwent D2 surgery, performed by the same surgeon in Zhongshan Hospital between 2000 and 2005, were consecutively enrolled in this study. Prior to surgery, no therapy was administered to any of the patients. All patients had a complete clinicopathological history recorded, including age, gender, tumor size, histological grade, American Joint Committee on Cancer (AJCC) tumor stage, depth of invasion, lymph node metastasis and distant metastasis (24,25). All patients presented with adenocarcinoma, and the median age of the patients at the time of diagnosis was 60 years (range, 30–80 years). The histological grade of the tumor was evaluated under a microscope and was categorized based on the degree of tumor differentiation, tumor necrosis and mitotic count according to the criteria of Enzinger and Weiss (26,27). Depth of invasion and lymph node metastasis were evaluated based on the National Comprehensive Cancer Network (NCCN) GC guideline (version 2011) (28). Follow-up time was calculated as the time of the initial surgery for the primary tumor until mortality or January 2013. The basic clinical information for all 94 patients is listed in Table I. Three of the patients presented positive for group no. 13 lymph node metastasis when enrolled and a D2 radical surgery was performed at that time according to the latest guidelines in 2011. These patients were labeled as exhibiting phase IV disease.

#### Tissue microarray (TMA) construction

From each patient, two cancerous and two non-tumor tissues (5 cm away from the tumor edge) were obtained for TMA construction and immunohistochemical (IHC) staining. Non-tumor/healthy tissues were defined as the paired gastric tissues that were 5 cm away from the tumor edge. Tissue sections (diameter, 1.5 mm; thickness, 4 µm) from archival, formalin-fixed, paraffin-embedded tissue specimens were mounted on poly-L-lysine-coated slides (Muto Chemicals, Tokyo, Japan). The sections were deparaffinized in xylene for 15 min, rehydrated in different concentrations of ethanol and then heated at 95°C for 5 min in 10 mM sodium citrate buffer (pH 6.0) in a microwave oven for antigen retrieval. Endogenous peroxidase was sequentially inactivated in 3% H_2_O for 15 min at room temperature.

#### IHC staining of the TMAs

For FAS staining, the sections were blocked in 3% normal donkey serum and subsequently incubated at 4°C overnight with monoclonal anti-FAS antibody (dilution, 1:50; #3180; Cell Signaling Technology, Inc., Danvers, MA, USA). Finally, the sections were stained with horseradish peroxidase (HRP)-conjugated donkey anti-rabbit immunoglobulin G (H+L) secondary antibody (Dako, Inc., Carpinteria, CA, USA).

For HER2 staining, the sections were first placed into a peroxidase-blocking reagent for 15 min. The primary antibody (dilution, 1:10; #2242; Cell Signaling Technology, Inc.) specific for HER2 was added and incubated at 4°C overnight. The sections were covered with Dako Envision+/HRP donkey anti-rabbit secondary antibody (Dako, Inc.) and incubated at room temperature for 30 min. Signal detection was performed using a Dako signaling amplification system (product no. K346811). The TMA was counterstained with hematoxylin, then dehydrated and mounted for better tissue structure identification. Certain other routine reagents were provided by the Department of Pathology, Zhongshan Hospital (Shanghai, China).

#### IHC score of FAS and HER2

All the IHC-stained slides were interpreted by one pathologist blinded to the sample identities. IHC scoring of FAS and HER2 was executed based on staining intensity and positivity. For each specimen, the staining intensity of FAS and cytoplasmic HER2 (cHER2) was scored as 0 for negative staining, 1 for weak intensity, 2 for moderate intensity and 3 for high intensity. The number of positive cells per section was categorized into three groups based on the percentage of positive cells: Group 1, <33%; group 2, 33–67%; and group 3, 68–100%, which were scored as 1, 2 and 3 respectively (positivity score). This method of positive scoring was demonstrated by Vandhana *et al* in 2011 (29). Total scores according to the semiquantitative immunoreactivity scoring (IRS) method were obtained by multiplying the staining intensity by the positivity score, leading to a range from 0 to 9. Ultimate scores (US) of FAS [US(FAS)] and cHER2 [US(cHER2)] were defined as the average of each of the two total scores for one tissue (two sections). Next, US(FAS) and US(cHER2) was categorized into two grades using the area under curve method for further analysis as follows: Weak staining for US(FAS), <6.75; strong staining for US(FAS), ≥6.75; weak staining for US(cHER2), <6; and strong staining for US(cHER2), ≥6.

**Figure 1.**
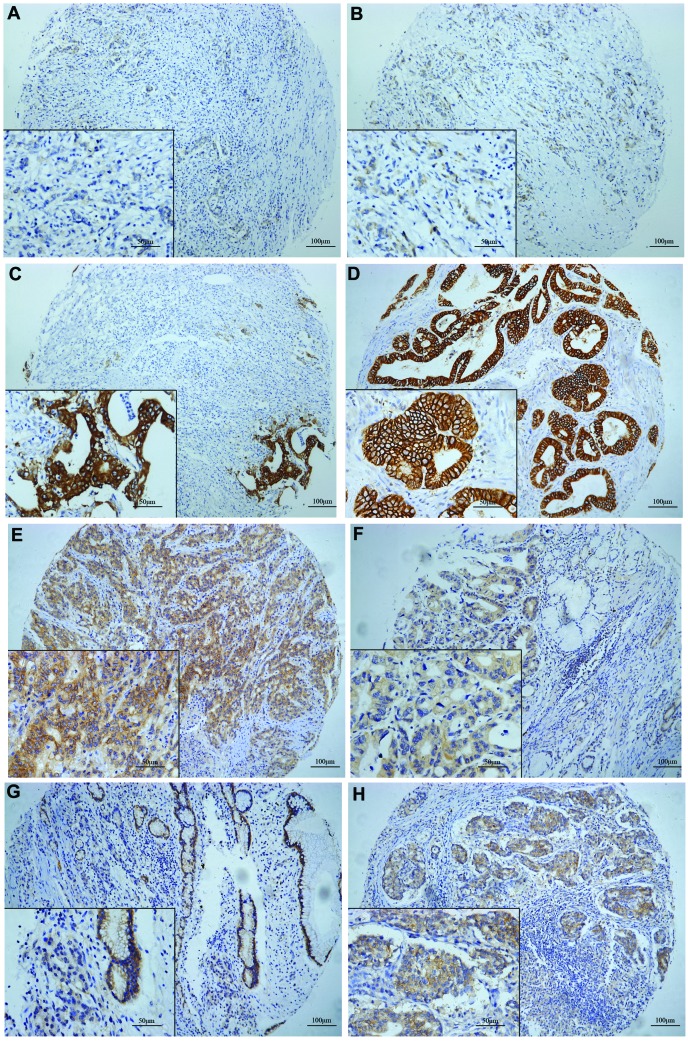
Fatty acid synthase (FAS) and human epidermal growth factor receptor 2 (HER2) expression in gastric cancer (GC) tissues. (A-C) Immunohistochemical staining of FAS in GC tissues. FAS was expressed in the cytoplasm according to different staining grades as follows: (A) Intensity, 1; positivity, 60%; (B) intensity, 2; positivity, 70%; and (C) intensity, 3; positivity, 90%. The cytoplasm and membrane stained for HER2. (D) Cytoplasmic (c)HER2: Intensity, 0; memranous (m)HER2: Intensity, 3; positivity, 95%; (E) cHER2: Intensity, 2; positivity, 90%; mHER2: Intensity, 2; positivity, 60%; (F) cHER2: Intensity, 2; positivity, 90%; mHER2: Intensity, 1; positivity, 20%; (G) cHER2: Intensity, 1; positivity, 60%; mHER2: Intensity, 0; (H) cHER2: Intensity, 3; positivity, 80%; mHER2: Intensity, 3; positivity, 20%. Neither FAS or HER2 were expressed in the nucleus. (A-C, E, G and H) Poor differentiation. (D and F) Good or moderate differentiation.

mHER2 IHC scoring was also performed based on intensity and positivity. The intensity of expression for each section was scored using four categories: 0+, meaning that there was no membranous staining in any of the tumor cells; 1+, meaning that there was membranous staining in <10% of the tumor cells with any intensity or in <30% of the tumor cells with weak intensity; 2+, meaning that there was staining in 10–30% of the tumor cells with moderate to strong intensity or staining in 30–50% of the tumor cells with weak to moderate intensity; and 3+, meaning that there was staining in >30% of the tumor cells with strong intensity or >50% of the tumor cells with any intensity. The average of the two scores for the same tissue was defined as the US(mHER2) ranging from 0 to 3. Tissues with a US(mHER2) score of ≤2 were classified as overexpressed (positive) (30). The schematic of the IHC staining for FAS and HER2 is shown in Fig. 1A–H.

#### Statistical analysis

The data were analyzed with GraphPad Prism 5 (GraphPad Software, Inc., La Jolla, CA, USA) for Windows. The paired t-test was used to compare the FAS and HER2 expression levels in the cancer tissues with those in the non-tumor tissues. Contingency Table analysis and χ^2^ tests were used to investigate the correlation between FAS and HER2 protein expression and clinical parameters, and the Fisher's exact test was used when qualified. The correlation between FAS and HER2 was determined mainly by using the Mann-Whitney rank test or unpaired t-test. The survival rate was estimated using the Kaplan-Meier method. Any difference in survival curves was compared by Wilcoxon test and a hazard ratio was obtained. P<0.05 was used to indicate a statistically significant difference.

## Results

### 

#### Overexpression of FAS in GC

**Figure 2.**
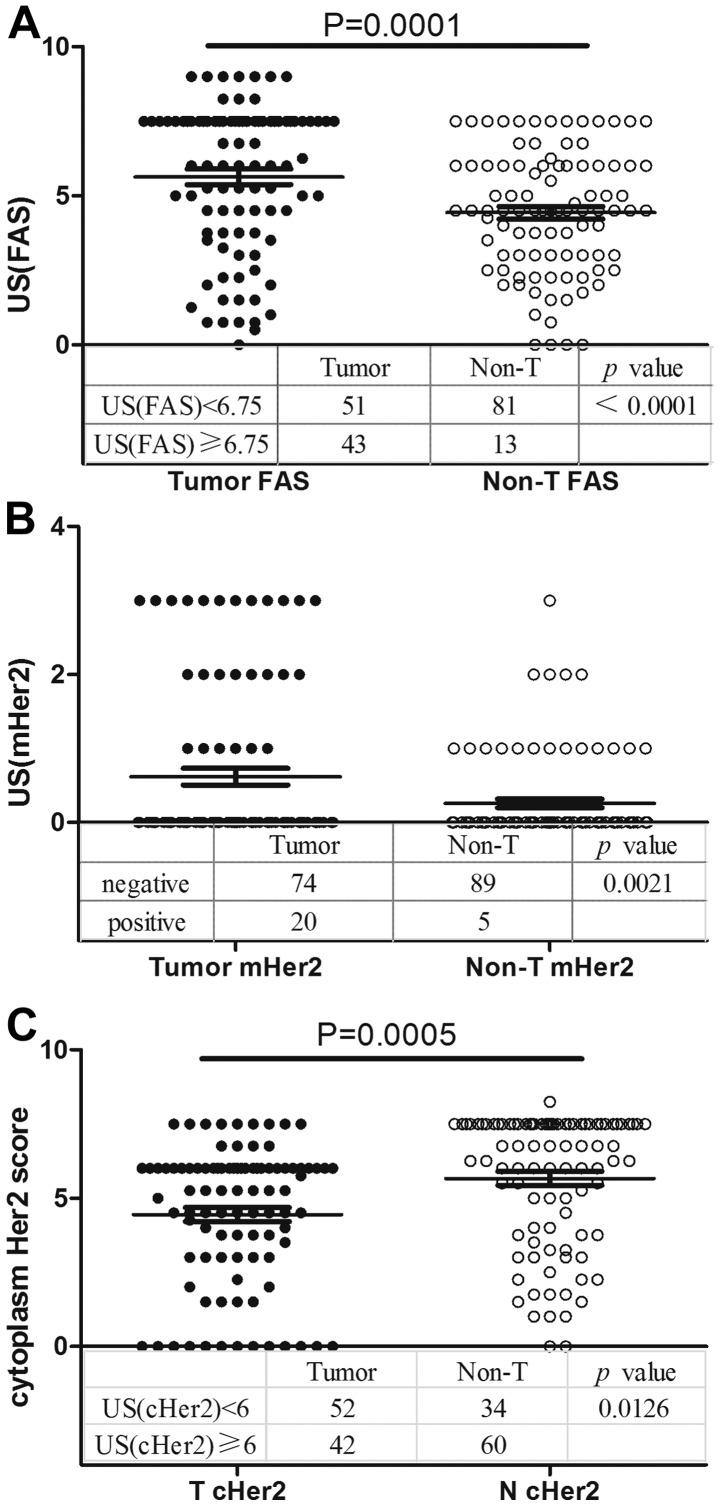
Expression of FAS and HER2 in tumor and non-tumor (non-T) tissues. (A) Comparison of FAS expression between tumor and non-tumor tissues. The gastric cancer (GC) tissues overexpressed FAS (5.633±0.510 vs. 4.431±0.423; P=0.0001; Mann-Whitney rank test; or P<0.0001; χ^2^ test). (B) Comparison of mHER2 staining status between tumor and non-tumor tissues. HER2 was significantly overexpressed in the GC tissues compared with the non-tumor tissues (P=0.0027; χ^2^ test). (C) Comparison of cHER2 expression between tumor and non-tumor tissues. Unexpectedly, in contrast to mHER2, cHER2 was underexpressed in the GC tissues (4.441±0.481 vs. 5.662±0.465; P=0.0005; Mann-Whitney rank test; or P=0.0126; χ^2^ test). FAS, fatty acid synthase; mHER2, membranous human epidermal growth factor receptor 2; cHER2, cytoplasmic HER2; US, ultimate score.

The FAS expression pattern in the 94 GC tissues and the adjacent non-tumor tissues was analyzed by TMA and IHC. FAS was expressed in the cell cytoplasm. A total of 54.3% (51/94) of the tumor tissues exhibited weak staining and 45.7% (43/94) exhibited strong staining, whereas these values were 86.2% (81/94) and 13.8% (13/94) in the non-tumor tissues (P<0.0001; χ^2^ test), respectively. FAS was overexpressed in the GC tissues compared with the normal tissues (5.633±0.510 vs. 4.431±0.423; P=0.0001, Mann-Whitney test; Fig. 2A).

#### Overexpression of mHER2 in GC

HER2 was expressed not only in the cytoplasm, but also on the membrane. Using classification variables, the significance of mHER2 staining scores was determined by χ^2^ test, and overexpression of mHER2 was present in 21.3% (20/94) of the tumors and 5.3% (5/94) of the non-tumor tissues [P=0.0021; relative risk, 1.762; 95% confidence interval (CI), 1.361–2.282; Fisher's exact test; Fig. 2B].

#### cHER2 is underexpressed in GC

cHER2 was found to be expressed in the GC and non-tumor tissues. In total, 44.7% of tumor tissues (42/94) exhibited strong staining (US(cHER2), ≥6) for cHER2, while 63.8% (60/94) of normal gastric tissues exhibited high expression levels of cHER2 (P=0.0126). Using the t-test to determine significance, the tumor tissues were shown to underexpress cHER2 compared with the non-tumor tissues (4.441±0.481 vs. 5.662±0.465; P=0.0005; Fig. 2C).

#### GC tissues exhibit a mutually strong correlation between FAS and mHER2

**Figure 3.**
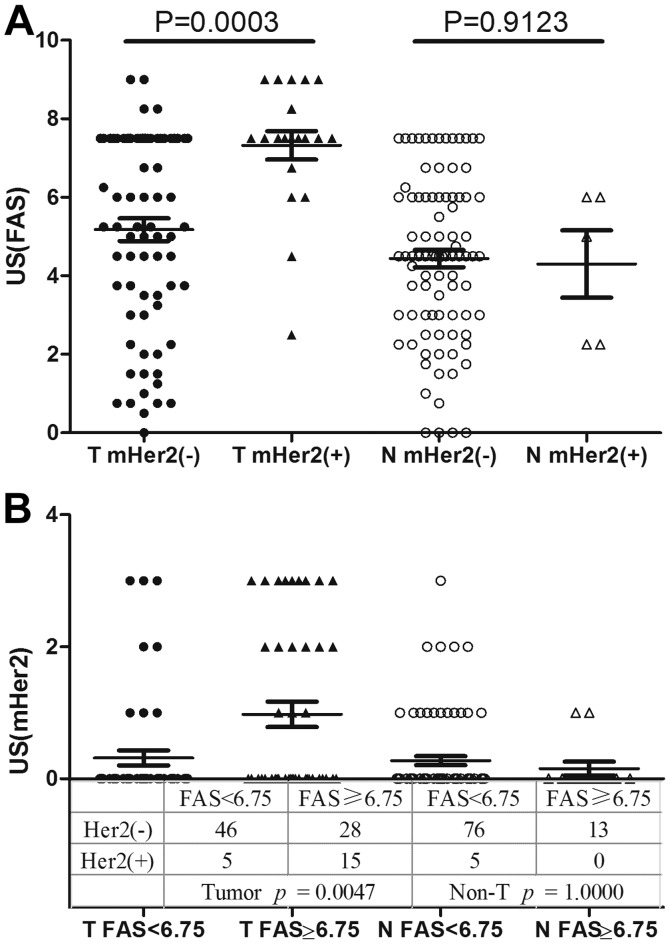
Co-expression pattern of FAS and mHER2 in gastric cancer tissues. (A) FAS expression shown according to mHER2 status (5.176±0.578 vs. 7.325±0.761; P=0.0003). In the HER2-positive group, FAS was overexpressed compared with the HER2-negative group, but this correlation only existed in the tumor tissues and not the normal tissues. (B) mHER2 expression shown according to different FAS status in the tumor tissues (0.3137±0.2284 vs. 0.9767±0.3886; P=0.0047; χ^2^ test). In non-tumor tissues, this phenomenon was not detected (P=1.0000). FAS, fatty acid synthase; mHER2, membranous human epidermal growth factor receptor 2; T, tumor tissue; N, non-tumor tissue; US, ultimate score.

There is a potential interaction between FAS and mHER2 in the signaling pathway mentioned in the introduction, and the present study further combined these two molecules to analyze the correlation between them. mHER2 expression was significantly upregulated in the FAS-strong group compared with its control, and the expression of FAS in the mHER2-positive group was greater than its expression in the mHER2-negative group. These results documented a potent and bidirectional significant correlation between FAS and mHER2 expression in the tumor tissues (a concordant expression pattern), but this pattern was not demonstrated in the non-tumor tissues (Fig. 3A and B).

#### A less differentiated state is associated with low cHER2 expression and is concordant with the expression of FAS and mHER2

The correlation between clinicopathological parameters, and the expression of FAS and HER2 was investigated. Clinical variables included age, gender, differentiation, AJCC stage, invasion depth, lymph node involvement, distant metastasis, tumor localization and tumor size. The results are listed in Table II. No significant correlations were detected between FAS and mHER2 expression. More significantly, a less differentiated state was associated with a concordant expression pattern [grade I+II vs. grade III; Fisher's exact test; P=0.0484; odds ratio (OR), 2.585; 95% CI, 1.084–6.167] and reduced cHER2 staining (P=0.0376; OR, 2.492; 95% CI, 1.076–5.772). In addition, female patients appeared to suffer a much higher risk of a concordant expression pattern compared with male patients (Fisher's exact test; P=0.0439; OR, 2.7595; 95% CI, 1.039–7.330).

#### Concordant expression of FAS and HER2 indicates a poor prognosis in GC patients

**Figure 4.**
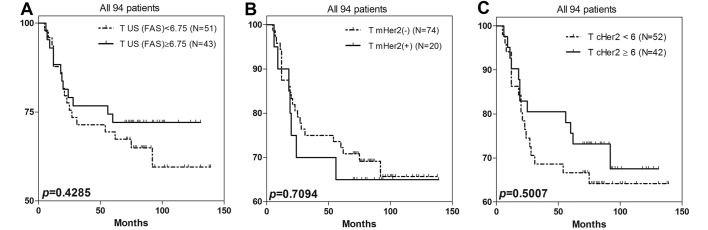
Survival analysis of all 94 GC patients. No correlation was detected between patient prognosis and the overexpression of FAS and HER2 when the data were analyzed separately. (A) Survival comparison by FAS in GC; P=0.4285; (B) survival comparison by mHER2 in GC; negative for a score of 0 and 1, positive for a score of 2 and 3; P=0.7094; (C) survival comparison by cHER2 in GC; weak for a score of <6, strong for a score of ≥6; P=0.5007. GC, gastric cancer; FAS, fatty acid synthase; mHER2, membranous human epidermal growth factor receptor 2; cHER2, cytoplasmic HER2; US, ultimate score.

**Figure 5.**
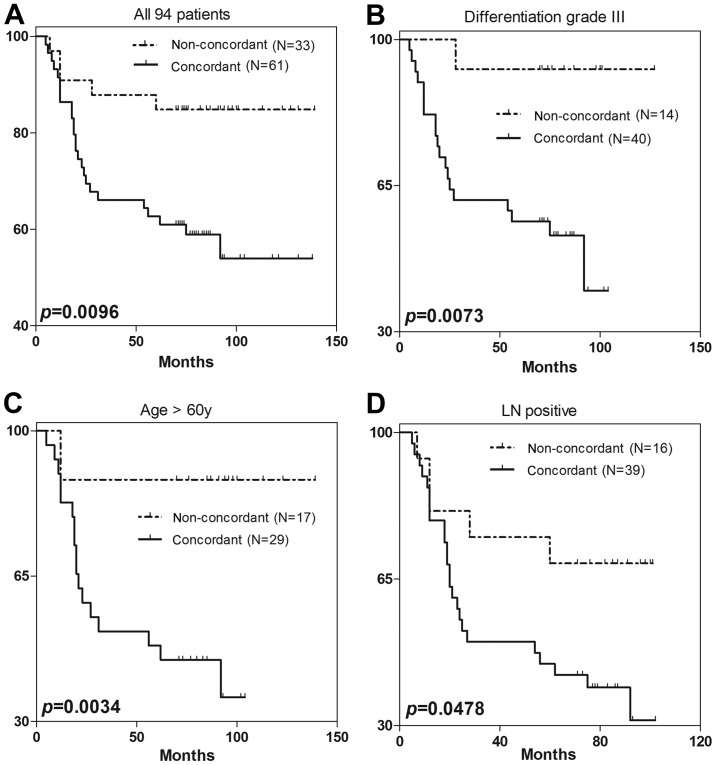
Prognostic analysis was conducted by combining FAS and mHER2 expression status. (A) There were significant differences in the survival outcomes in the two groups (Wilcoxon test; P=0.0096; hazards ratio, 2.647; confidence interval, 1.267–5.532). The group with concordant expression of FAS and HER2 showed a worse outcome. When stratified by different (B) tumor differentiation grade, (C) patient age and (D) lymph node (LN) metastasis, the prognostic significance could still be obtained. FAS, fatty acid synthase; mHER2, membranous human epidermal growth factor receptor 2.

Although it has been demonstrated that the *in vitro* overexpression of FAS and mHER2 commonly predicts a poor survival rate (20–23), the present data showed no significant overall survival difference between the groups classified by FAS (P=0.4285; Fig. 4A), mHER2 (P=0.7094; Fig. 4B) or cHER2 (P=0.5507; Fig. 4C). However, when combining mHER2 and FAS together, and analyzing the data in the two groups as concordant or non-concordant, a prognostic difference was found between the groups. The survival curves of the patients are presented in Fig. 5A (Wilcoxon test; P=0.0096; HR, 3.2801; 95% CI, 1.5781–6.8176). When stratified by tumor differentiation, age and node metastasis, patients with concordant expression still showed a worse prognosis in differentiation grade III, elder and positive lymphatic metastasis patients (Fig. 5B–D).

## Discussion

The treatment of GC has improved during the last few decades with regard to the surgical skills and tumor targeted strategies, however, the general outcome for GC patients remains inadequate, and various studies have been conducted on gastric carcinogenesis and novel targeted molecules (2,31–33). FAS, a crucial synthesizer of LCFAs, is an enzyme that is involved in the synthesis of normal lipids and the development of cancer (5). FAS overexpression and increased activity represents one of the most recurrent phenotypic variations in cancer cells. A number of growth factors and their receptors, including HER2, have materialized as major contributors to the overexpression of FAS. However, the mechanisms ultimately responsible for tumor-associated FAS overexpression are not completely understood (5). In the present study, a potent and bidirectional correlation between FAS and mHER2 expression and the potential value for predicting patient outcome was primarily demonstrated in the GC patients. These novel findings may indicate an important role for these two combined molecules in gastric carcinogenesis and tumor invasiveness, and the potential benefits for future targeted therapy.

The present data showed that FAS and mHER2 were overexpressed in 45.7% (43/94) and 21.3% (20/94) of the GC tissues, respectively. The expression of these proteins in the cancer tissues was higher than their expression levels in the non-tumor tissues.

As shown in former studies, the positivity of FAS ranges from 50% to >80% in various types of tumors (15,34–38), apparently overexpressed in comparison with paired non-tumor tissues. In 2002, Kusakabe *et al* demonstrated the overexpression of FAS in GC tissues by IHC methods (39), and a following study was conducted to investigate the potential function of FAS in gastric carcinogenesis *in vitro* (40). HER2 as a membranous molecule has been reported to be overexpressed in various percentages of GC patients according to different studies (41–43). A study involving 1,414 GC patients showed that 17% of GC tissues would overexpress mHER2 (44). The present data was also consistent with these results. The mechanism of FAS and HER2 overexpression in cancer has been the subject of several studies; however, the total representation remains far from understood.

It has been hypothesized that there must be a potential correlation between FAS and mHER2 (45), with a number of studies confirming this fact, for example, in various tumors, including breast (22,46,47), ovary (20) or oral (23,48) cancer, with mHER2 and FAS upstream and downstream molecules of the PI3K and MAPK pathways. In normal human tissues, this type of correlation has not been found between FAS and mHER2. The present results showed that in GC tissues, the expression of FAS may be elevated along with mHER2 overexpression, and vice versa. This interaction appeared to serve as a positive-feedback pathway that can mutually regulate the expression of FAS and mHER2. This result showed that FAS and mHER2 were definitely correlated in GC, which was consistent with studies in other tumors (20–23).

The mechanisms involved in the mutual regulation between mHER2 and FAS have been mainly revealed. mHER2 activates the FAS gene promoter through the PI3K and MAPK signaling pathway, and finally elevates FAS expression. Moreover, mHER2 can directly activate FAS protein by its intracellular phosphorylation domain (22). Alternatively, HER2 gene expression and HER2 protein activity can be modulated through the concentration changes of acetyl-CoA and malonyl-CoA that are regulated by FAS (5,49). In addition, as the key enzyme of *de novo* synthesis, FAS can increase the stability of mHER2 by the formation of a domain known as a lipid raft, located on the membranes (46). These mechanisms ultimately construct a positive-feedback pathway between mHER2 and FAS.

The present study did not find any correlation between the clinical information and the expression of mHER2 and FAS. Generally, mHER2 is considered to be highly correlated with intestinal GC (Laurén type) (50–54). However, information about Laurén type intestinal GC was not included in the present study. The reasons for this correlation between mHER2 and FAS, and the mechanisms behind it, remain to be elucidated. Other clinical parameters, including differentiation grade, tumor-node-metastasis stage and tumor size, were not confirmed to correlate with mHER2 expression. FAS has been found to be highly expressed in well-differentiated GC tissues compared with poorly-differentiated GC tissues, and it also appears to function in the early stage of gastric carcinogenesis (39). This deduction was not statistically evident in the present study when the correlation between FAS expression and tumor differentiation was analyzed, but 55.0% (22/40) of grade I and II GC tissues demonstrated overexpression of FAS, and this ratio was 38.9% (21/54) in grade III tissues. So there may be a decreasing trend of FAS expression along with worsening tumor differentiation. In the generation of the majority of tumors, FA synthesis is a highly activated process to supply enough phospholipid and enzymes for the rapid proliferation of tumor cells. However, there is no standard FAS scoring system to evaluate its IHC staining level, which has shown variations and discrepancies among different studies (3,4,39,40,55.

Currently, controversy remains with regard to the prognostic value of mHER2 in GC patients (43,44,56,57), and former observational studies and the ToGA trial do not have a uniform conclusion to this issue (2,43,58–60). However, a number of recent studies have shown that elevated expression levels of mHER2 are associated with tumor invasion and a poor prognosis (56,61). However, the present study did not find that mHER2 exhibited prognostic value in GC patients, which may be due to several factors. GC only showed mHER2 positivity in ~20% of the patients, but the sample capacity of the study was too limited to detect the potential and probable significance. Moreover, fluorescence *in situ* hybridization is commonly considered to be the gold standard in the evaluation of mHER2 expression (62), and other IHC methods may have a bias tendency. However, the consistency of these two methods is ~93.5%, as proposed by the ToGA trial (2). Moreover, the Herceptin standard was recommended by NCCN to evaluate the IHC staining of mHER2, and the present study used the criteria proposed by Chung *et al* in 2005 (30), thus it suggested that bias and deviations inevitably exist in spite of their good concordance. Finally, mHER2 is commonly overexpressed more simply in intestinal GC, but the Laurén type of GC in the present study was unknown, which may be a confounding factor in prognostic analysis, since a diffuse type definitely indicates an inferior prognosis. Therefore, after the patients were stratified by differentiation grade, it was found that in the poorly-differentiated groups, positive mHER2 expression significantly indicated a poor prognosis (P=0.0153), which may have resulted from a certain elimination of perplexing factors. On the other hand, this result showed that tumors with mHER2 expression have a higher capability for invasion and metastasis, which is in disagreement with a former study (63). Therefore, the function and regulation of HER2-mediated pathways in gastric carcinogenesis are intricate and complex.

The value of FAS in predicting GC prognosis is not yet confirmed, although FAS has been considered to be correlated with the prognosis of various tumors, such as non-small cell lung carcinoma (37), melanoma (38) and soft-tissue sarcomas (64). It has been a more commonly accepted fact that FAS does not associate with the prognosis of GC patients. The present data did not find any significant correlation between FAS and patient survival, which is in agreement with a former study (39). In 2009, Dowling *et al* reported that FAS inhibitors could apparently induce the apoptosis of GC cells *in vitro* and depress tumor formation in mice, which to some extent reflect the potential roles of FAS in gastric carcinogenesis (40). This requires further investigation in more depth. However, notably, the present study found that the concordant expression group suffered a much worse prognosis compared with the non-concordant group, when FAS and mHER2 were combined together in a survival analysis. The five-year overall survival rates were 62.7 and 84.8% in these two groups. Moreover, in the elder patients (>60 years), the females and the patients with grade-III differentiation, concordant expression still acted as a predictor of a poor prognosis. It has been reported *in vitro* that prostate cells expressing FAS and androgen receptor (another activator of the PI3K-Akt pathway) can form invasive adenocarcinomas in immunodeficient mice, however, cells that expressed only FAS did not (65). Thus, we can hypothesize based on present data, that in non-tumor tissues, the positive-feedback pathway of mHER2-FAS is not activated, and no correlation was found between them. However, this pathway could be activated in a certain stage of gastric carcinogenesis and would promote gastric cell proliferation. Therefore, the patients that present with FAS/mHER2 concordant expression may have a worse prognosis, due to the activation of the mHER2/FAS pathway.

However, not all GC patients presented with a pattern of concordant expression, and a large proportion of the patients showed non-concordant expression of FAS and mHER2, which may be a presentation of GC heterogenicity or result from other unknown mechanisms involved in the process of gastric carcinogenesis. Nevertheless, mHER2 and FAS are simultaneously modulated by various molecules in a complicated process, and more intensive and detailed cytological experiments are required to confirm or investigate this theory.

cHER2 expressed in GC has seldom been investigated in depth. Unexpectedly, the present study found that cHER2 was significantly overexpressed in non-tumor tissues compared with tumor tissues. In addition, cancerous tissues with good differentiation (grade I+II) showed a higher percentage of cHER2 overexpression in comparison to poorly-differentiated cancerous tissues [57.5% (23/40) vs. 35.2% (19/54); P=0.0376; Fisher's exact test]. It appears that the expression of cHER2 has a tendency to increase with GC progression. Therefore, cHER2 may play completely different roles from mHER2, in normal and tumor tissues. With tumor progression, mHER2 increases and cHER2 decreases, and complicated mechanisms must be involved in this process.

Currently, the complications that arise from the use of HER2-targeted therapy in GC treatment are not fully understood, but the experience and information gained from HER2-targeted breast cancer therapy indicates that a large number of HER2-positive breast cancer patients are primarily resistant to anti-HER2 drugs, and almost all of the patients will have drug-resistant tumors following HER2-targeted therapy (66–69). However, it has been proved *in vitro* that the combination of anti-FAS and anti-HER2 targeted therapy will decrease the resistance to HER2 inhibitors through various mechanisms (21,22,70,71). Therefore, the present study preliminarily offers evidence for the feasibility of using combined detection of FAS and mHER2 in GC patients, which appears most significant in mHER2-negative patients and mHER2-positive patients who are resistant to HER2-targeted therapy. Also, from the present results, we further surmise that this promising method will be of more use in the personalized treatment of GC patients who are >60 years old, female and who have tumors with poor differentiation. However, a great deal of research is required in order to achieve this goal, and more cytological and genetic studies are required.

In conclusion, FAS and mHER2 are overexpressed in GC tissues, with a strong association with each other. The present study showed a concordant expression pattern of FAS and mHER2 in GC tissues, while there was no association between these two markers in healthy, adjacent non-tumor tissues. The two proteins are linked to the same signaling pathway (MAPK and PI3K/Akt), and their expression is associated with a poor prognosis for GC patients. cHER2 was also found to be underexpressed in GC tissues.

## Figures and Tables

**Table I.** Complete clinical information of 94 gastric cancer patients.

Characteristics	Value
Gender, n (%)	
Male	61 (64.89)
Female	33 (35.11)
Age, years	
Median	60
Range	30–80
Histological type, n (%)	
Adenocacinoma	94 (100.00)
Other	0 (0.00)
Presentation, n (%)	
Initial	94 (100.00)
Recurrent	0 (0.00)
Size, cm	
Median	3
Range	0.3–10
Differentiation, n (%)	
I	6 (6.38)
II	34 (36.17)
III	54 (57.45)
Metastasis, n (%)	
Negative	91 (96.81)
Positive	3 (3.19)
AJCC stage, n (%)	
I	30 (31.91)
II	18 (19.15)
III	43 (45.74)
IV	3 (3.19)

AJCC, American Joint Committee on Cancer.

**Table II.** Correlation of FAS and mHER2 protein expression in gastric cancer with the clinicopathological characteristics of 94 patients.

		US(FAS)			mHER2			cHER2			Combination of FAS and mHER2		
					
Characteristic	n	Weak	Strong	P-value	–	+	P-value	Weak	Strong	P-value	Concordant	Non-concordant	P-value
Gender				0.0867			0.6078			0.5176			0.0439
Male	61	29	32		49	12		32	29		35	26	
Female	33	22	11		25	8		20	13		26	7	
Age, years				0.5348			0.0443			0.2130			0.8294
Median		58	62		59	65		57.5	63		59	60	
Range		30–77	43–80		30–77	36–80		30–77	40–80		30–80	36–73	
<60	48	28	20		42	6		30	18		32	16	
≥60	46	23	23		32	14		22	24		29	17	
Histological grade				0.1454			0.2158			0.0376			0.0484
I+II	40	18	22		29	11		17	23		21	19	
III	54	33	21		45	9		35	19		40	14	
TNM stage			0.4160			0.3183			0.5408			0.1995	
0+1+2	48	24	24		40	8		25	23		28	20	
3+4	46	27	19		34	12		27	19		33	13	
Depth of invasion				1.0000			0.1163			0.8324			0.1198
T0+1+2	35	19	16		31	4		20	15		19	16	
T3+4	59	32	27		43	16		32	27		42	17	
LN metastasis				0.4055			0.6126			0.5342			0.1892
N0	39	19	20		32	7		20	19		22	17	
N1+2+3	55	32	23		42	13		32	23		39	16	
Distant metastasis				1.0000			1.0000			1.0000			1.0000
M0	91	49	42		71	20		50	41		59	32	
M1	3	2	1		3	0		2	1		2	1	
Tumor size, cm				1.0000			0.3171			0.4073			1.0000
<3	38	21	17		32	6		19	19		25	13	
≥3	56	30	26		42	14		33	23		36	20	
Localization				0.9592			0.9689			0.1973			0.3279
Up	10	5	5		8	2		3	7		5	5	
Median	44	24	20		35	9		27	17		27	17	
Down	40	22	18		31	9		22	18		29	11	

LN, lymph node; US, ultimate score; FAS, fatty acid synthase; mHER2, membranous human epidermal growth factor receptor 2; cHER2, cytoplasmic HER2; TNM, tumor-node-metastasis.
